# Barriers to diverse clinical trial participation in Duchenne muscular dystrophy: Engaging Hispanic/Latina caregivers and health professionals

**DOI:** 10.1186/s13023-024-03209-7

**Published:** 2024-05-21

**Authors:** Norah L. Crossnohere, Nicola B. Campoamor, Eric Camino, Erin Dresnick, Daphne Oluwaseun Martschenko, Viana Rodrigues, Susan Apkon, Alexis Hazlett, Dhruv Mittur, Priscilla E. Rodriguez, John F. P. Bridges, Niki Armstrong

**Affiliations:** 1 Division of General Internal Medicine, Department of Internal Medicine, The Ohio State University College of Medicine, Columbus, OH USA; 2 Department of Biomedical Informatics, The Ohio State University College of Medicine, Columbus, OH USA; 3 Parent Project Muscular Dystrophy, Washington, DC USA; 4 Center for Biomedical Ethics, Department of Pediatrics, Stanford Medicine, Stanford, CA USA; 5 Department of Physical Medicine and Rehabilitation, University of Colorado School of Medicine, Aurora, Colorado USA; 6 Patient partner, Parent Project Muscular Dystrophy, Washington, DC USA; 7 Diversity Inclusion Advocacy Manager, EveryLife Foundation for Rare Diseases, Washington, DC USA

**Keywords:** Duchenne muscular dystrophy, Diversity, equity and inclusion, Clinical trial as topic

## Abstract

**Background:**

Despite the increasing availability of clinical trials in Duchenne muscular dystrophy, racial/ethnic minorities and other populations facing health disparities remain underrepresented in clinical trials evaluating products for Duchenne. We sought to understand the barriers faced by Hispanic/Latino families specifically and underrepresented groups more generally to clinical trial participation in Duchenne.

**Methods:**

We engaged two participant groups: Hispanic/Latino caregivers of children with Duchenne in the US, including Puerto Rico, and health professionals within the broader US Duchenne community. Caregiver interviews explored attitudes towards and experiences with clinical trials, while professional interviews explored barriers to clinical trial participation among socio-demographically underrepresented families (e.g., low income, rural, racial/ethnic minority, etc.). Interviews were analyzed aggregately and using a thematic analysis approach. An advisory group was engaged throughout the course of the study to inform design, conduct, and interpretation of findings generated from interviews.

**Results:**

Thirty interviews were conducted, including with 12 Hispanic/Latina caregivers and 18 professionals. We identified barriers to clinical trial participation at various stages of the enrollment process. In the initial identification of patients, barriers included lack of awareness about trials and clinical trial locations at clinics that were less likely to serve diverse patients. In the prescreening process, barriers included ineligibility, anticipated non-compliance in clinical trial protocols, and language discrimination. In screening, barriers included concerns about characteristics of the trial, as well as mistrust/lack of trust. In consent and recruitment, barriers included lack of timely decision support, logistical factors (distance, time, money), and lack of translated study materials.

**Conclusions:**

Numerous barriers hinder participation in Duchenne clinical trials for Hispanic/Latino families and other populations experiencing health disparities. Addressing these barriers necessitates interventions across multiple stages of the clinical trial enrollment process. Recommendations to enhance participation opportunities include developing clinical trial decision support tools, translating prominent clinical trials educational resources such as ClinicalTrials.gov, fostering trusting family-provider relationships, engaging families in clinical trial design, and establishing ethical guidelines for pre-screening potentially non-compliant patients.

**Supplementary Information:**

The online version contains supplementary material available at 10.1186/s13023-024-03209-7.

## Background

There is a growing awareness that diversity and representation matter in clinical trials. One-fifth of drugs reviewed by US Food and Drug Administration (FDA) demonstrated some differences in response across race/ethnicity [1]. People with the same condition may experience it differently, as well as respond to treatments differently, for a number of reasons including social and environmental factors, personal health attitudes, and an individual’s unique genomic profile [2, 3]. The lack of appropriate representation of the patient population in clinical trials results in studies with limited generalizability. As a result, regulatory groups and clinical trial funders have indicated that trial participants should reflect the age, race, and ethnicity of people experiencing the condition [4–6]. Ensuring appropriate representation of racial minorities and other populations facing health disparities is a priority area for federally funded research [7]. 

Duchenne muscular dystrophy is a rare neuromuscular disorder primarily affecting males. It is characterized by premature loss of ambulation (typically 10–14 years of age) and death (typically 20–40 years of age) [8]. Duchenne occurs due to a pathogenic variant on the dystrophin gene which can be either inherited or de novo. Its incidence is estimated at 1 in 5000 live male infants [9, 10]. Current therapeutic regimes are centered on glucocorticoids, exon skipping therapies, and a newly approved gene therapy. In the past decade people with Duchenne have benefited from substantial improvements in length and quality of life, in large part due to advancements in supportive care. As of writing, 20 clinical trials are actively recruiting individuals with Duchenne in the US [11]. In addition to observational studies, these trials evaluate a range of therapies including those aimed to restore or replace dystrophin, improve and protect muscle, improve heart function, and reduce inflammation and fibrosis [12]. 

There is great unmet need in the Duchenne community, and the need to rapidly advance drug development may have historically worked against efforts to foster more diverse clinical trial participation. Racial/ethnic minorities are estimated to be underrepresented in clinical trials for Duchenne [13]. Numerous burdens hinder participation in Duchenne trials generally, including those that are technical, financial, psychosocial/emotional, and related to informed consent [14]. Hispanic/Latino populations are underrepresented across clinical trials nationally, with estimates indicating that Hispanic/Latinos make up nearly 19% of the US population but only 11–16% of clinical trial participants [15, 16]. This disparity is even greater in the context of Duchenne trials despite Hispanic/Latinos having a potentially higher prevalence of Duchenne muscular dystrophy than other ethnic groups [17]. How barriers and burdens impact trial participation among underrepresented groups has not been studied but is essential to improving equitable drug development and access to clinical trials in Duchenne.

In this study, we sought to explore the barriers to participation in clinical trials for Duchenne muscular dystrophy reported by Hispanic/Latino families living in the US, as well as those observed for other underrepresented groups more generally. We sought to explore these barriers across multiple levels, including the individual patient, provider, health care, clinical trial, and social systems. This work can inform an understanding of obstacles hindering the participation of diverse patients in clinical trials for Duchenne and help identify solutions to address these barriers.

## Methods

### Stakeholder engagement

We engaged an advisory group to shape study design, consistent with the research team’s prior work with the Duchenne community [18–26]. We assembled an advisory group (*n* = 12) of patients with Duchenne, caregivers, members of industry, and individuals trained in clinical trial design and management, ethics, and diversity, equity, and inclusion (DEI) (see acknowledgements). They provided input on the development of the study, including the selection of caregiver populations, recruitment approach, and content of interviews. Advisory group members met once individually with members of the study team to provide their individual insight to the research program and met twice as a larger group during the planning of the study. They also provided input on study materials such as interview guides via email. Advisory board members and other stakeholders in the Duchenne community also contributed to data analysis through in-person activities (see ‘data analysis’).

Hispanic/Latino populations were chosen as the focus of the study population based on the input of the advisory group. Specifically, the advisory group was compelled given epidemiological evidence that Hispanic/Latino boys may have a higher prevalence of Duchenne as compared to other racial/ethnic groups [17] and given that they might also experience linguistic barriers to participate in trials. Additionally, several members of the advisory group were champions for Hispanic/Latino populations and were able to help facilitate interviews with eligible caregivers.

### Participants and recruitment

As we sought to understand multi-level barriers associated with clinical trial participation in Duchenne, we recruited individuals with a broad range of experiences in relation to Duchenne clinical trials including caregivers, physicians, clinical trialists, nurses and research nurses, and clinical trial coordinators. Given the dearth of research on clinical trial participation for Hispanics/Latinos with Duchenne, we committed to focusing all caregiver interviews within this population. Recruitment of health care and research professionals was not restricted by race and ethnicity, nor by the race or ethnicity of the patient population they treat.

We recruited caregivers through various sources including in-person recruitment at a clinic for Spanish-speaking patients with Duchenne, distribution of information about the interview study to a Spanish-focused patient advocacy group, and word of mouth. We recruited health care professionals through email distribution to a list-serve for individuals affiliated with Certified Duchenne Care Centers. Individuals were eligible to participate if they were: over 18 years of age, residing in the US, and English or Spanish speaking. For caregiver participants, additional eligibility included that they be a caregiver of a person with Duchenne under the age of 18 and self-identify as Hispanic/Latino. Caregivers were eligible to participate regardless of whether their child had participated in a clinical trial. For health professionals, additional eligibility included that they have at least two years’ experience in referring, enrolling, or conducting clinical trials with Duchenne patients. Caregiver participants were compensated with a $100 gift card for their participation. Health professionals were not compensated for their time.

### Data collection

Interviews were conducted in person or over the Zoom platform and recorded [27]. Verbal consent was collected prior to initiating the interview. The interview guide was developed to elicit multi-level barriers to clinical trial enrollment and participation and shaped to align with domains posed in the Health Equity Implementation Framework [28]. One team member took field notes during the interview, and interviews were audio recorded and transcribed. The interviews were semi-structured in nature and interviews were completed in English or Spanish, depending on the preference of the participant. Spanish interviews were led by a heritage speaker.

### Data analysis

Data in this study was analyzed thematically in an iterative process that used both inductive and deductive coding. We inductively developed codes that reflected barriers expressed by caregiver and health care professional participants (NLC, NBC). We then assigned these codes to corresponding stages of clinical trial recruitment and retention including the identification of patient population, prescreening, screening/consent, and enrollment. Field notes were used to generate initial codes. Transcript review was used to refine codes and identify representative quotes. The study observed the Standards for Reporting Qualitative Research [29]. The lead researcher on the study (NLC) is a patient-centered researcher with a long-standing research portfolio exploring patient experiences in Duchenne. She acknowledges these experiences may influence data interpretation. To maintain reflexivity, she regularly reflected on her assumptions and engaged in discussions with members of the study advisory board.

Data credibility was established through prolonged investigator (NLC) engagement with the data over a six-month period of time [30]. The credibility of data coding and interpretation was also reinforced through checking of the data with members of the advisory group. This included assessing the dependability of results through comparing findings from the current study to those of other clinical trial centers across the country. To do this, we presented results at two national Duchenne care and research meetings attended by health professionals and parents, where we compared our findings to the experiences expressed by other individuals over the course of the meeting. All study procedures were reviewed by The Ohio State University IRB (2023E0220; 2023E0470).

## Results

We interviewed 12 Hispanic/Latina caregivers (6 in Spanish), and 18 professionals (Tables 1 and 2). Caregiver participants were all female, and mostly mothers (*n* = 10) of children with Duchenne. Professionals included neurologists, research nurses, coordinators, and investigators. Interviews lasted on average 48 min. Health professionals indicated that they saw diverse patient populations and reported caring for rural, low socioeconomic status, non-English speaking, international, and Hispanic/Latino patients.

**Table 1:** Caregiver demographic characteristics (*N* = 12)

Caregiver	No. (%)
**Female**	12 (100)
**Age**, mean (range)	41 (23–71)
**Ethnicity: Hispanic/Latino**	12 (100)
**Marital status** ^**1**^	
Married / Domestic partnership	4 (67)
Divorced	1 (17)
Single	1 (17)
**Region**	
Southwest	5 (8)
West	2 (17)
Southeast	2 (8)
Puerto Rico	3 (25)
**Highest level of education: Less than BA**	6 (50)
**Employed full-time** ^1^	3 (50)
**Relationship to person with Duchenne**	
Mother	10 (83)
Grandmother	1 (8)
Sister	1 (8)
**Very comfortable completing medical documents** ^2^	
In Spanish	10 (83)
In English	4 (33)
**Child with Duchenne**	
**Age**, mean (range)	11 (3–16)
**Age at diagnosis**, mean (range)	4.3 (0–8)
**Ambulatory**	6 (50)
**Participated in clinical trial**	1 (8)
**Health Insurance: public program**	8 (67)

^1^ Missing for 6 respondents

^2^ Not mutually exclusive, sum to greater than 100%

**Table 2:** Health professional characteristics (*N* = 18)

Health professionals	No. (%)
**Female**	14 (78)
**Professional role**	
Neurologist	8 (44)
Research nurse	4 (22)
Research coordinator	2 (11)
Nurse	1 (6)
Physiatrist	1 (6)
Researcher	1 (6)
Social worker	1 (6)
**Region**	
West	7 (39)
South	6 (33)
Midwest	4 (22)
Northeast	1 (6)
**Clinical trials conducted**	18 (78)
Industry sponsored	13 (72)
Investigator led	5 (36)

**Fig. 1:**
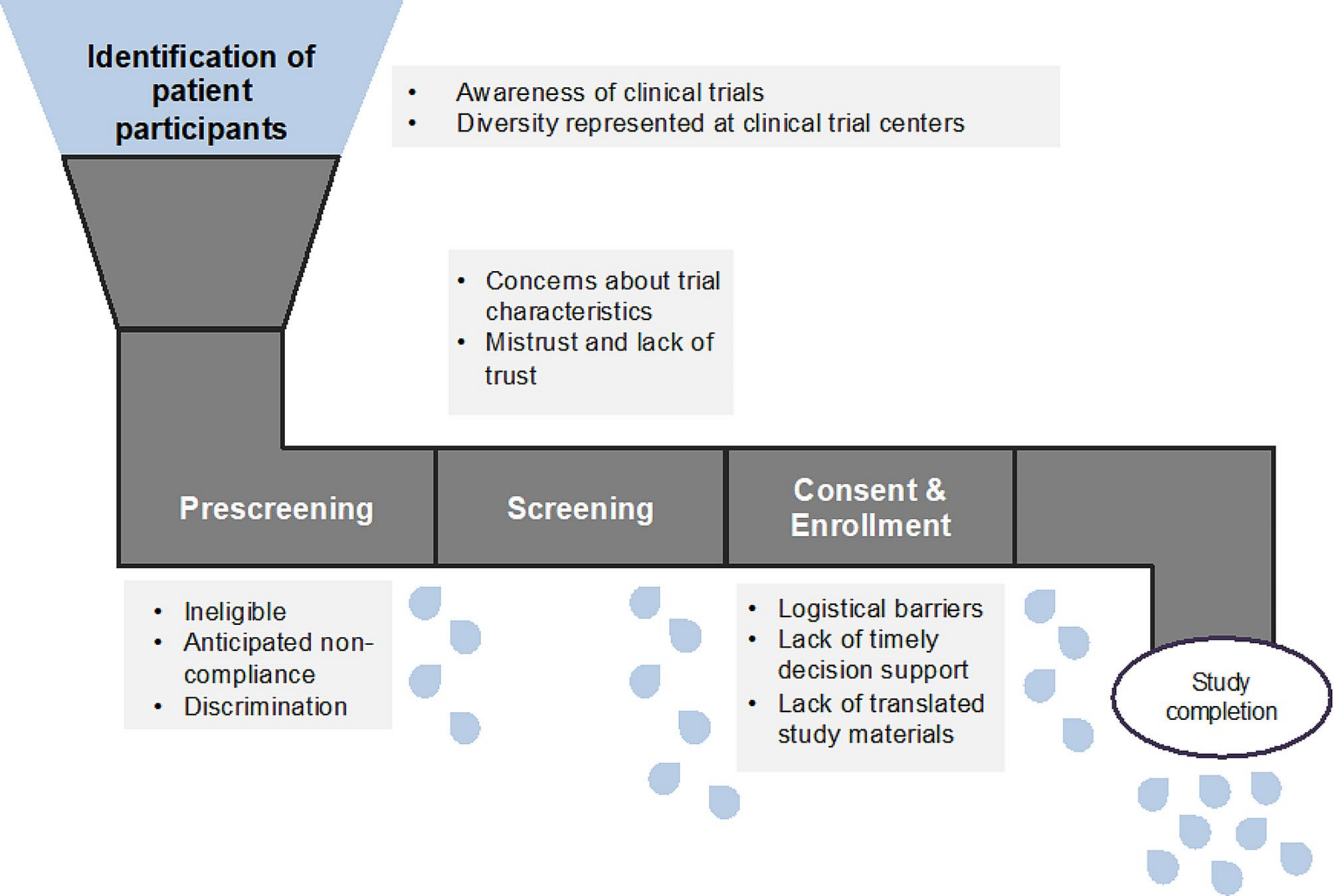
“Leaky pipe”: Loss of diverse participants across all stages of a clinical trial

Below we present the barriers to clinical trial enrollment and participation across four main stages: Identifying potential participants, prescreening, screening/consent, and enrollment. These barriers across stages are visualized in Fig. 1. Quotes exemplifying the barriers are included in Table 3.

**Table 3:** Illustrative quotes describing barriers to participation

Barriers		Example Quote			
**Identification of patients**					
Awareness	*There’s 2 kind of main streams of patients, right? The families that are very well in the know… and then there’s the patients that you just kind of, you mentioned clinical trials to, as they, as you notice that they are coming in, and that they qualify. ‘I’ll think about that.’ And it just kind of depends on where they’re at, you know. There’s a lot of stuff going on in the family.* – Nurse coordinator				
Diversity of clinical trial center	*It’s about 8% [Black families] that come to clinic. I’m in a state that’s got about, based on the last census, 23–24% Black population. So, if they’re not coming to clinic, they’re certainly not going to come get into trials.* – Neurologist				
**Prescreening**					
Ineligibility	*We tested him for the antibodies and everything. He always tested negative. So, he was a good candidate [for gene therapy]. Unfortunately, it’s taken too long, and he became non-ambulatory.* –Caregiver				
Anticipated non-compliance	*Because there’s so much structure in a trial, their compliance with everything gets better because they just have that framework.* – Nurse				
Discrimination	*We have had points in time where we do feel slightly discriminated towards because we’re Hispanic. It hasn’t been often, and it hasn’t been [his primary] doctors…whenever something like that has happens, we’ve, you know, pointed it out to someone we trust, and it’s changed or like it’s help.* – Caregiver				
**Screening**					
Characteristics of the trial	*There’s one [clinical trial] going on now…it would require from my son to be able to go through like an MRI, for like an hour and a half following instructions… and that’s just impossible.* – Caregiver				
Mistrust or lack of trust	*Some of the parents are very hesitant because they might be here illegally, even if the child is born here and is…a citizen, they want to stay under the radar.* – Clinical nurse				
**Consent and Enrollment**					
Lack of timely decision support	*Me dijeron de esos estudios, pero la verdad me entré en pánico porque no tuve la información necesaria y no hablo inglés, solo español, entonces no tuve la oportunidad en entender muchas cosas porque, primeramente, es mi primer niño con esta enfermedad y para mí era muy desconocida [DMD]… pues como que eso me hico decir que no.* – Caregiver *They told me about clinical trials and like I told you, I started to panic. I didn’t have the necessary information for the study, and I don’t speak English, only Spanish, so I didn’t have the opportunity to understand many things because firstly this is the only child I have with this disease, and I do not know the information surrounding it [the clinical trial or DMD]… so it made me decide to say no. –* Caregiver				
Logistical (distance, money, time)	*“We have people 3 hours away one way. So, if you have an appointment at 10 in the morning, but you have to be here at 8:30 to do your EKG, and it’s a 3-hour drive and it’s morning traffic, and it’s in the middle of the winter. You’re getting up at like 4 in the morning to leave.*” – Nurse coordinator				
Lack of translated study materials	*Solo me dijeron que me iban a mandar un paquete en español explicándome todo en lo que él podría hacer en el estudio, pero no recibí nada. Por eso es porque he tenido esa desconfiancita en los ensayos clínicos y no poner el niño [su hijo]. – Caregiver* *They told me that they would send me a packet in Spanish explaining everything …but in the end, I didn’t receive anything. Since then, I have always had that mistrust with clinical trials and enrolling my son in them. –* Caregiver				

### Identification of patient participants

Many health professionals described a lack of *family awareness of clinical trials* as a barrier to participation. One professional described that, “*There’s two kind of main streams of patients, right? The families that are very well in the know… and then there’s the patients that you just kind of, you mentioned clinical trials to, as they, as you notice that they are coming in, and that they qualify, [and say] ‘I’ll think about that.’”* Caregivers and providers indicated that lack of awareness was not because of interest, but rather because of difficulty in finding information about trials, especially if they had low English proficiency. Several remarked that platforms to search for clinical trials, like ClinicalTrials.Gov, are not available in Spanish, which impacted their ability to identify ongoing studies.

Not having a *diverse patient population* treated at clinics that serve as trial sites was a large barrier to enrollment. Reflecting on their own experience, one professional noted that the main pool of patients in a clinical trial is pulled from the preexisting patient population seen at the clinic where the trial is located. They went on to describe that, “*it’s about 8% [Black families] that come to clinic. I’m in a state that’s got about…23–24% Black population. So, if they’re not coming to clinic, they’re certainly not going to come get into trials.”* Other professionals reflected that their location was not particularly diverse, and so that even if their patient population reflected their surrounding community, their participants would still be relatively homogeneous.

### Prescreening

In prescreening, members of the clinical trial team conduct an initial review of patients to identify potential eligible participants for a given study. Health professionals described various approaches to prescreening, spanning simple medical record review to full study team meetings discussing patient and family characteristics. In the prescreening process, *ineligibility*, due to factors such as type of genetic variant, age, mobility status, or prior clinical trial participation, were commonly cited reasons why patients would not be approached about clinical trials. In reflecting on waiting for a clinical trial including a gene therapy to become available for their son, one caregiver reflected, “*Unfortunately, it’s taken too long, and he became non-ambulatory…so he’s out of [consideration for] that.”* Several professionals also commented that ineligibility was also sometimes a linguistic factor, and that non-English speaking participants were immediately excluded from studies if the trial site did not have translated study materials or multi-lingual clinical evaluators.

Some health professionals reported that they would go on to approach every patient/family who was not blatantly ineligible, with one stating, *“If they’re eligible, they have a right to know.”* Other professionals reported that they would also consider factors such as whether the patient was a good candidate for a clinical trial based on characteristics such as their anticipated non-compliance with study procedures, their family environment, and history of missed appointments. Making prescreening decisions to not approach patients based on *anticipated non-compliance* was described by some health professionals as a safety issue, who described the importance of routine monitoring for patients receiving experimental therapies. Others indicated that failing to offer a clinical trial to a family based on anticipated non-compliance reflected an ingrained bias among clinical trial teams which disproportionately resulted in the exclusion of patients from families with fewer resources and social support.

Both caregivers and professionals indicated that *bias & discrimination* impacted opportunities for clinical trial participation. In reflecting on visiting a clinician who her son had been referred to as a candidate for a clinical trial, one caregiver reflected that, *“we have had points in time where we do feel slightly discriminated towards, because we’re Hispanic. It hasn’t been often, and it hasn’t been [his primary] doctors…whenever something like that has happens, we’ve, you know, pointed it out to someone we trust, and it’s changed or like it’s helped.”* In this case, the family shared their experience with their primary physician, who recommended that they not return to this health care provider. The cost of doing so, however, was that the family did not participate in the trial. A professional also described the role of discrimination, particularly in unconscious biases. They reflected, that “*it’s us [health care providers], we’re the problem.”* Bias was particularly problematic when trying to establish appropriate language. Providers noted that sometimes in their effort to communicate clearly, they would change their selection of wording, moving from highly specific terms to more general ones that they thought would be more easily captured by translation services. Caregivers indicated that these sorts of changes sometimes diluted the underlying message.

### Screening

Multiple caregivers reported that their decision to participate in a clinical trial would be influenced by *characteristics of the clinical trial* itself which were learned about during the screening appointment. Some caregivers indicated that the discomfort of invasive procedures such as muscle biopsies were too burdensome for their child. One caregiver reflected that she makes choices about her son’s trial participation, *“depending on what the procedure is, how invasive, and also the side effects. Because my son…he has autism, too, so it’s hard for him to process. I don’t want to traumatize him.”* Several health care professionals indicated that they had experiences where families would refuse participation in clinical trials unless they were gene-therapy related, though none of the caregivers indicated this was an explicit barrier.

*Mistrust or lack of trust* was another barrier to participation that arose during the screening process. In reflecting on experiences with a general pool of patients, rather than those who were Hispanic/Latino specifically, several professionals indicated that some families had a mistrust of health care systems. This sentiment was not widely expressed by Hispanic/Latino caregivers interviewed. Rather, caregivers indicated that while they sometimes did not trust a particular provider, their perception of health care systems was generally positive. After telling interviewers about an instance wherein a provider made condescending comments to the caregiver and her family about the extra work the study would be for the research team because the family requested translated materials, the caregiver went on to clarify that she did not feel that way about all doctors. For some, apprehension also results from concern about legal status. A clinical nurse described that “*some of the parents are very hesitant because they might be here illegally, even if the child is born here and is…a citizen, they want to stay under the radar.”* Caregivers themselves did not express this sentiment, however.

### Consent and enrollment

Both caregivers and professionals indicated that *logistical challenges* were a barrier to clinical trial participation. Examples of logistical challenges cited included the expenditure of time, money, and resources needed to participate in clinical trials. These barriers were significant; “*We have people 3 hours away one way,”* reflected a professional. These challenges existed even for families who technically lived near centers offering clinical trials; and some caregivers described that they still would spend time in transit, require them to take off work, and cause their child to miss school. In reflecting on logistical challenges, health care providers also indicated that these burdens were likely even more burdensome on families who were low income, potentially exacerbating disparities. Several Spanish-speaking caregivers reported typically bringing another family member to their child’s clinic appointments to be an informal translator. To ask that family member to also attend clinical trial sessions would impose these burdens on them as well.

Several health professionals raised that a challenge in recruiting underrepresented groups, including those who may be less aware of or not actively seeking clinical trials, was the *lack of timely decision support*. Among underrepresented groups, professionals reported there was a need for more time and resources to inform participation. For instance, families may need to be approached about the trial several times, reminded about potential participation, and may take several months before ultimately deciding about the trial. One professional described a scenario wherein they had wanted a family to participate in a trial, but they had needed more time to consider. By the time they agreed, the slot had been filled by a different patient. As a result, the professional described the family as feeling jilted and resentful towards the clinical team. The provider went on to reflect that, “*trials prioritize families that jump on the trial, rather than those that ask more questions.”* Caregivers also indicated that they needed time to review and make decisions about trial participation, and that not having this affected their decision to participate. One caregiver reflected that, “*They told me about those studies, and like I told you, I started to panic. I didn’t have the necessary information for the study, and I don’t speak English…not having all the necessary information to know what the positives and negatives of the studies were, it made me decide to say no.”*

Several health professionals also described that their sites had experienced push-back and delays in getting *translated study materials* from sponsors and approval from IRBs. They reflected that protocols often do not accommodate the use of in-clinic translation to complete study activities. One caregiver described an experience wherein the clinical trials group told them that *“they would send me a packet in Spanish explaining everything …but in the end, I didn’t receive anything. Since then, I have always had that mistrust with clinical trials and enrolling my son in them*.” Not all health professionals reported challenges in receiving Spanish-translated materials, however. To proactively overcome issues and delays related to translation, a research coordinator at one clinic which primarily served Hispanic/Latino families indicated that, *“our standard is that if we agree to do a clinical trial, we want everything translated in Spanish from the get-go.”*

## Discussion

This study qualitatively explored barriers to clinical trials among underrepresented and health disparity populations with Duchenne, with a focus on Hispanic/Latino families. Barriers to participation in trials spanned multiple stages of the clinical trial process. Our findings show that each stage of the clinical trial recruitment and enrollment process posed stage-specific barriers to diverse participation in clinical trials. Improving diverse representation in clinical trials in Duchenne can be advanced by patching a leak at any of these points but can be fully achieved by addressing them all.

Previous research has explored barriers and facilitators of clinical trial participation in the Duchenne muscular dystrophy patient community. In 2018, Peay et al. surveyed parents of children with Duchenne, Becker, or SMA (*n* = 203) about their attitudes towards clinical trials [31]. Interest in participating in trials was high, and the largest concern was receiving a placebo. Although some participants in the current study expressed a disutility to receiving a placebo, it was not a pervasive phenomenon, and expressed by caregiver rather than professional participants. Caregivers in the current study were more likely to express concern for the potential side effects of experimental therapy, as well as express concern for the procedures conducted as a part of the clinical trial, such as muscle biopsy or even MRI.

Additionally, a prior interview study of clinicians and parents about their experiences in clinical trials for pediatric neuromuscular disorders indicated that clinicians perceived themselves to have more influence on decision-making than attributed by parents [32]. Parents indicated that they decided to participate in a trial before the consent process and equated not enrolling their child to doing harm. The current study of Hispanic/Latino families found this not to be the case; rather, families expressed that characteristics of the trial such as use of placebos, biopsies, MRIs, etc., of which were discussed during the screening and consent process, would influence their decisions to participate.

The inclusion of Spanish-language interviews was a strength of the study, and allowed researchers to understand the experiences of Duchenne families in the US who speak limited English. These families represent a particularly vulnerable group as they not only experience the challenges of their rare disease, but also may experience disadvantage because of their and/or their family’s lack of fluency in English. Our exploration was limited to the perspectives of caregivers and professionals in the US. There are also several limitations of this study. First, we experienced substantial difficulty in identifying and recruiting Hispanic/Latino caregivers to participate in this study. Although we observed saturation of major themes, the study may still have benefited from the inclusion of more caregivers, especially those who had children who had participated in clinical trials. The most successful approach for recruitment was to approach families while they were at a multidisciplinary care center, in part relying upon the established trust between the families and their care provider. However, these discussions also tended to be shorter and less in-depth than those conducted via Zoom. Additionally, we did not collect the race/ethnicity of healthcare professionals, which could have been used to better contextualize healthcare professionals’ own lived experiences.

### Key recommendations to improve diverse representation in Duchenne clinical trials

While the current study focused on exploring barriers to participation in trials, recommendations to help alleviate several of these burdens emerged throughout. Many of these barriers have specific actions which can be taken, a list of these is included in Table 4. Below we highlight what we see as the key recommendations to improving diversity in clinical trials. The first recommendation is to develop and disseminate Spanish-language resources regarding clinical trials for Duchenne. Both caregiver and professional participants in this study noted a lack of resources to support Spanish-language searching for clinical trials. Several English-speaking caregivers and many professionals indicated that families search for trials through ClinicalTrials.Gov. However, Spanish-speaking participants commented on the lack of Spanish-language resources about clinical trials, and specifically that ClinicalTrials.Gov was not available in Spanish. In addition to translating ClinicalTrials.Gov to additional languages, efforts might also consider improving the readability and decreasing the grade-level of ClinicalTrials.Gov content.

**Table 4:** Barriers and corresponding recommendations

Stage	Barrier		Example action(s) to reduce barriers	Group to implement actions
Identification of patients	Families unaware of clinical trials generally		Discuss trials routinely as a part of patient care	Clinical care provider
	Families unaware of specific clinical trial opportunities		Develop and disseminate Spanish-language resources regarding clinical trials, such as translated patient recruitment materials and translated ClinicalTrials.gov*	Sponsors; Government; Patient groups
	Lack of diversity among patients seen at clinical trial centers		Compare clinical trial site patient demographics to local demographics as a component of site selection	Sponsors
	Clinical trial sites not located in diverse areas		Evaluate clinical demographics in the area surrounding a clinic as a part of site selection	Sponsors
Prescreening	Patient ineligibility based on unmodifiable patient factors (age, mobility, previous medication use)		Maintain a culture of research by providing individuals options to participate in registry-based studies, longitudinal studies, etc.	Sponsors; Trial site
	Patient ineligibility based on potentially modifiable factors (behavior, cognition)		Have frank discussions about expectations of clinical trial, and use educational tools to help family and patients make informed decisions about whether they have the capacity to modify factors to participate	Trial site
	Clinician, researcher biases in prescreening		Establish internal protocols for prescreening and presenting trials to families; Discuss with family reasons for anticipated non-compliance; Develop ethical guidelines for the prescreening of potential clinical trial participants*	Trial site; Patient groups
Screening	Medical burdens of trial (e.g., biopsy, side effects, hospitalizations, MRI)		Be forthcoming with burdens of trial and provide modifications whenever possible; allow patient care teams to access the clinical trial test results of their patients reduce redundant testing	Sponsors
	Mistrust and poor experiences with providers		Proactively foster trust between caregivers and providers, and provide clear avenues for patients to report discriminating experiences*; Implement provider trainings to reduce bias and unconscious bias	Trial site
	Mistrust among healthcare and research systems		Increase diverse patient and caregiver engagement in the design of clinical trials for Duchenne*	Sponsors; Patient groups
	Lack of readily-translated materials		Provide all materials in languages present at clinic prior to initiating trial	Sponsors
	Low-quality medical interpretation		Have in-person certified medical interpreter services with experience in clinical research translation	Sponsors; Trial site
Consent and enrollment	Lack of timely decision support		Develop and implement clinical trial decision support tools*	Sponsors
	Far distance/time to travel		Increase flexibility in clinical trial design; co-design clinical trial schedule to coincide with other patient care	Sponsors; Trial site
	Need for childcare of other children		On-site childcare	Sponsors; Trial site
	Financial burdens of trial		Pre-pay expenses rather than reimburse	Sponsors

* Indicates key recommendation

The second recommendation is to develop and implement clinical trial decision support tools. We observed a particular need for clinical trial decision support among caregivers who indicated that they relied upon clinical trialists for information about a study. This need was also expressed by professionals, who indicated that families typically underrepresented in clinical trials often needed more time and support to facilitate their decision making than other families. Decision support could include structured decision aids with bolt-on dimensions to reflect the unique set of benefits, risks, and processes of a given clinical trial. Alternatively, it could be a structured and shared decision-making discussion template. Decision support tools should be delivered in the preferred language of the patient.

A third recommendation, observed throughout interviews, was that trust between caregivers and providers often helped to overcome barriers to clinical trial participation. We heard from caregivers of experiences wherein they decided not to participate in a trial because they did not trust the provider. More generally, having a trusting relationship with health care providers may help families to fully understand the purpose, risks, and potential benefits of clinical trials. There are specific activities that providers can do to promote trust with their patients. These include: actively listening to the questions, concerns, and experiences of patients, using language that is clear but does not dilute the underlying medical message, being timely in communication, and demonstrating empathy and compassion for the social and medical experiences of patients [33]. Trust encourages open dialogue, allowing families and patients to express concerns or ask questions about the trial. Diversity and representation in the health care and clinical trial teams enables better cultural competence and can increase trust [34]. 

A fourth recommendation is to increase engagement of underrepresented patients and caregivers in the design of research for Duchenne. Participant engagement in research is characterized as the meaningful involvement of patients, caregivers, and other stakeholders throughout the research process and including in activities such as identifying research questions, selecting study designs, and advising on the conduct and dissemination of research [35]. Participant engagement provides an opportunity to improve the quality and relevance of medical research, including in Duchenne specifically, which can lead to increased trust of trials [36]. Including the patient/family perspective helps to challenge assumptions held by researchers and clinicians, shifting the focus of research onto topics that are more patient-centered. Established frameworks provide guidance on how to facilitate this engagement in research specifically to promoting diversity, equity, and inclusion [37]. 

A fifth recommendation is to develop recommendations to advise on the ethical prescreening of potential clinical trial participants. Professionals in this study expressed divergent perspectives on their approaches for prescreening, with some indicating that any eligible patients should be approached about trials, and others being more discerning, particularly if there were concerns about the family’s anticipated compliance in the study. More robust consideration of both perspectives and how they impact both individual families and diversity, equity, and inclusion concerns in clinical trials would help guide clinical trial groups in prescreening patients.

## Conclusions

In this study we identified barriers to clinical trial participation among Hispanic/Latino families specifically and underrepresented populations across the US more generally. We found that challenges to participating in clinical trials arose at all stages of the clinical trial process, necessitating intervention across various stakeholders including providers, clinical trial staff, clinical trial groups, and other stakeholders. Practical recommendations to address these challenges include developing multi-lingual resources and decision support tools, increasing engagement to foster trust, and developing ethical screening guidelines. These recommendations are proposed to facilitate more inclusive and equitable enrollment in Duchenne clinical trials.

### Electronic supplementary material

Below is the link to the electronic supplementary material.

Supplementary Material 1

## Data Availability

Data from this study are not openly available due to reasons of sensitivity and are available from the corresponding author upon reasonable request. Data are located in controlled access data storage at The Ohio State University.
